# Investigation of the relationship between immune checkpoints and mismatch repair deficiency in recurrent and nonrecurrent glioblastoma

**DOI:** 10.3906/sag-2010-166

**Published:** 2021-08-30

**Authors:** Emel ÇAKIR, İsmail SAYGIN, Mustafa Emre ERCİN

**Affiliations:** 1 Department of Pathology, Faculty of Medicine, Karadeniz Technical University, Trabzon Turkey

**Keywords:** Glioblastoma, mismatch repair, programmed cell death protein ligand 1 (PD-L1), programmed cell death protein 1 (PD-1), microsatellite instability

## Abstract

**Background/aim:**

Microsatellite instability tests and programmed cell death-1 (PD-1)/programmed cell death ligand-1 (PD-L1) in the immune checkpoint pathway are the tests that determine who will benefit from immune checkpoint inhibitor therapy. We aimed to show the expression of DNA mismatch repair proteins and PD-1/PD-L1 molecules that inhibit immune checkpoints, to explain the relationship between them, and to demonstrate their predictive role in recurrent and nonrecurrent glioblastoma.

**Materials and methods:**

We analyzed 27 recurrent and 47 nonrecurrent cases at our archive. We performed immunohistochemical analysis to determine expressions of PD-1, PD-L1, and mismatch repair proteins in glioblastoma. We evaluated the relationship between these two group and compared the results with the clinicopathological features.

**Results:**

The mean age of diagnosis was significantly lower in recurrent glioblastoma patients. Median survival was longer in this group. We found that PD-L1 expression was reduced in recurrent cases. Additionally, recurrent cases had a significantly higher rate of microsatellite instability. Loss of PMS2 was high in both group but was substantially higher in recurrent cases.

**Conclusion:**

The presence of microsatellite instability and low PD-L1 levels, which are among the causes of treatment resistance in glioblastoma, were found to be compatible with the literature in our study, with higher rates in recurrent cases. In recurrent cases with higher mutations and where immunotherapy resistance is expected less, low PD-L1 levels thought that different combinations with other immune checkpoint inhibitors can be tried as predictive and prognostic marker in GBM patients.

## 1. Introduction

Glioblastoma (GBM) is the most common primary brain tumor with a poor prognosis (47.1%) [1]. GBMs are fatal tumors with a median survival time of 12 months. Approximately 3%–5% of cases live more than 3 years [2]. Many studies are showing the importance of genetic susceptibility, exogenous factors, age and clinical parameters at the time of diagnosis, as the reason for aggressive behavior [3–6]. Other important factors are surgical procedures and multimodal treatment options. Immunotherapy is a promising treatment method that shows a synergistic effect with radiotherapy [7].

Immune checkpoints can be grouped into two main groups as immunostimulating and blocking. The main molecules that inhibit immune checkpoints are the cytotoxic t-lymphocyte-associated protein-4 (CTLA-4), programmed death-1 (PD-1) receptor, and its two ligands (PD-L1 and PD-L2). These molecules block control signals that lead to the T cell response against the tumor. New treatments are aimed at inhibiting PD-L1 on the tumor cell or PD-1 receptor on the T cell which will produce an antitumoral response. These treatment agents are called immune checkpoint inhibitors (ICIs). CTLA-4, one of the major inhibitory molecules in GBMs, is released from T cells, binding to its ligands (CD80, CD86) reduces the activation and proliferation of effector T cells and increases the activation of regulatory T cells (Treg) in the GBM microenvironment [8,9]. 

PD-1 is released from T cells and other immune cells. One of its ligands, PD-L1 is found in Tregs in the GBM microenvironment, tumor-associated macrophages, and other cells in the tumor microenvironment, including tumor cells [8,10]. The predictive markers in PD-1/PD-L1 antibody therapy are mainly the number of cytotoxic T-lymphocytes inside tumor tissues and the expression level of PD-L1 in cancer cells [11,12]. In extensive studies, PD-L1 expression levels in GBMs were found between 61%–88% [13–15]. High PD-L1 mRNA expression level was found to be associated with shorter overall survival in glioma patients [12,16,17]. 

Treating recurrent GBMs is more difficult and resistance develops more frequently to treatment. These tumors have been treated several times (radiotherapy and temozolomide) and therefore contain a greater number of mutations. Due to the presence of potential new antigens, these tumors are assumed to be more suitable for the recognition and attack of the immune system. In contrast to the hypothesis that the tumor assumes that it will increase the release of PD-L1 with the immune-escape mechanism developed to protect itself from stronger immune response, in some studies, this rate was found to be significantly lower in recurrent tumors [13,14,18]. 

PD-1/PD-L1 expression, tumor mutation load, and DNA mismatch repair (MMR) defects are thought to be related to the treatment response. Some clinical studies revealed that defective MMR is associated with clinical responses to immune checkpoint inhibitors (ICI) [5,19]. Tumors with high microsatellite instability (MSI) and high immunogenicity benefit from immunotherapy more and have a better clinical course. Therefore, it is recommended to use MSI status as a marker for response to PD-1/PD-L1 blockade in cancer patients [20]. 

In the last 6 years, significant results have been obtained in immunotherapy in various tumors (melanoma, renal cell cancer, lung cancer, head, and neck cancers) with anti-PD-1/PD-L1 antibodies. While the response to PD-1 inhibitors is significantly high in lymphoma subtypes (87% in Hodgkin’s lymphoma), this rate ranges from 15% to 40% in solid organ cancers [21,22]. There are several studies showing that PD-1 inhibition increases the antitumor responses and survival rate on animal glioma models [23,15]. Especially combined immune checkpoint blockade resulted in 100% long survivors [23]. However, there is insufficient clinical evidence to support its effectiveness in GBM patients. There are some “case reports” in the literature showing that anti-PD-1 therapy (nivolumab) have significant therapeutic effects on GBM patients [19,24,25].

## 2. Material and methods

### 2.1 Patients and clinical information

In our study, 74 cases (recurrent and nonrecurrent GBM) diagnosed in our department between 2007–2019 were selected. Twenty-seven of these cases showed recurrence. Hypercellular tumors including palisading necrosis and vascular endothelial proliferation were accepted as ‘original tumor’. Diffuse necrosis, vascular hyalinization, gliosis, and the presence of rare atypical cells were accepted as ‘radiotherapy effect’. High-grade glioma with mitosis and minimal evidence of radiation effect is defined as ‘recurrent tumor’ [26]. The first resection materials of the recurrent cases were defined as “Group 1”, the 2nd resection materials were “Group 2” and the nonrecurrent cases were defined as “Group 3”. When calculating rates in recurrent cases, cases were considered “positive” if any of the cases in group 1 or 2 were positive. The results of recurrent and nonrecurrent GBM patients were compared. In addition, in recurrent cases, the expression rates in the 1st and 2nd resection materials were compared. For each case, the patient’s age at the time of diagnosis, sex, time for recurrence, and survival were recorded. Clinical information was obtained from patient files on the computer.

### 2.2 Immunohistochemical study

**Figure 1:**
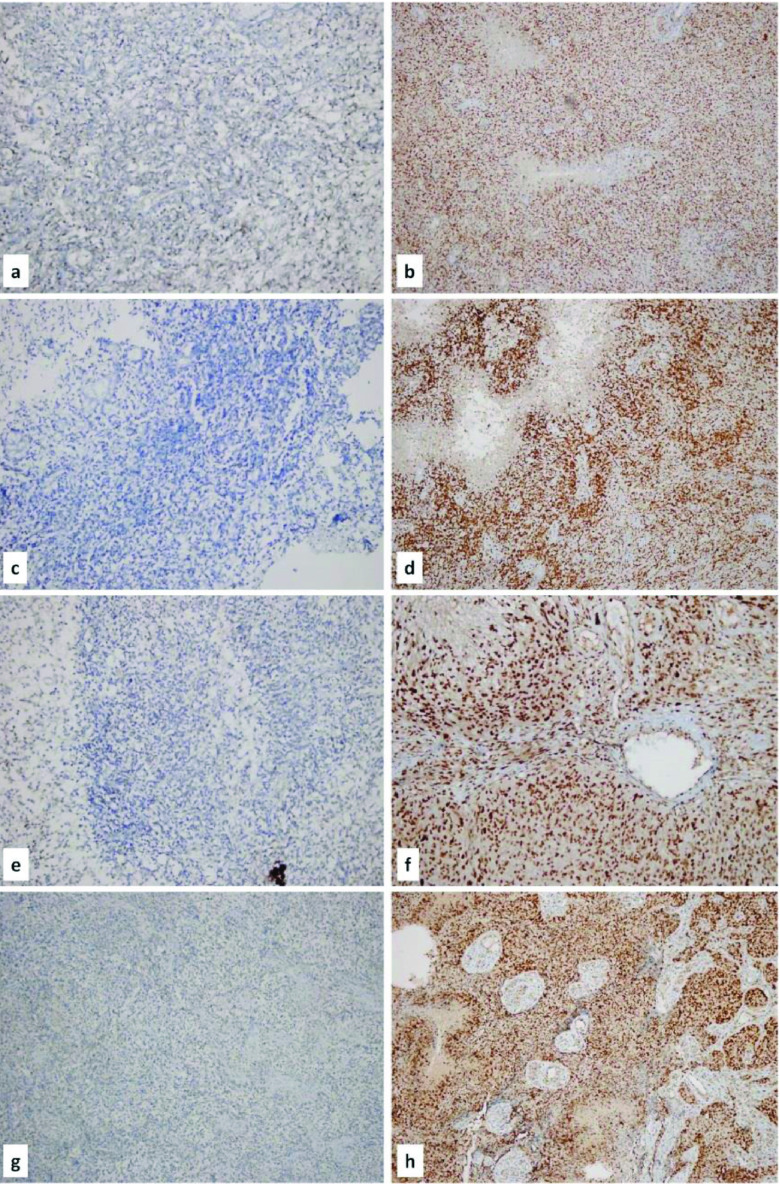
Expressions of MMR proteins.

Immunohistochemically, the relationship between MLH1 (MutL Homolog 1), MSH2 (MutS Homolog 2), MSH6 (MutS Homolog 6), and PMS2 (PostMeiotic Segregation increased 2) results and immune checkpoint inhibitors PD-1 and PD-L1 were examined and compared with the clinicopathological features. Sections stained with immunohistochemical antibodies were examined under a light microscope (Olympus BX50). Normal colon tissue was used as a control of the immunohistochemical MLH1, MSH2, MSH6, and PMS2 markers. Expressions of MMR proteins were evaluated as follows: Nuclear staining in more than 80% neoplastic cells was accepted as score 4; 51%–80% staining score 3; 10%–50% staining score 2; less than 10% staining score 1 and no nuclear staining score 0 [27]. Expressions of MMR proteins were shown in Figure 1 (Figure 1: a) Loss of nuclear staining with MLH1 (×200), b) Nuclear staining with MLH1 (×100), c) Loss of nuclear staining with MSH2 (×200), d) Nuclear staining with MSH2 (×100), e) Loss of nuclear staining with MSH6 (×200), f) Nuclear staining with with MSH6 (×200), g) Loss of nuclear staining with with PMS2 (×100), and h) Nuclear staining with PMS2 (×100)). Loss of nuclear expression of one or more MMR proteins was accepted as deficient mismatch repair [28].

SP263 clone of the PD-L1 antibody was used. Diffuse fibrillar/membranous staining in tumor tissue was evaluated. According to previous studies, no staining in nonnecrotic tumor tissue was accepted as score 0; <25% staining score 1; 25%–50% staining score 2; 50%–75% staining score 3 and > 75% staining score 4 [13]. Membranous staining in epitheloid tumor cells was defined as (+) if > 5% staining in tumor cells as in previous studies [13].

**Figure 2:**
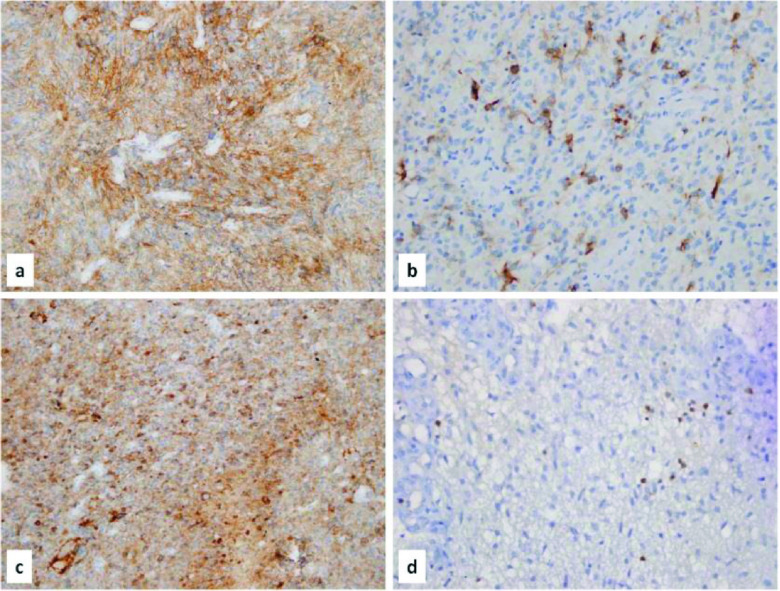


PD-1 expression was seen in lymphocytes in tumor tissue, and perivascular space. It was scored as sparsely, moderately and intensively according to the staining rates in large magnification (200×–400×). The staining patterns of PD-L1 and PD-1 were shown in Figure 2 (Figure 2: a) Diffuse fibrillary PD-L1 staining in tumor matrix (×200), b) Membranous PD-L1 staining on tumor cells (×400), c) Diffuse fibrillary and membranous PD-L1 staining (×200), d) PD-1 staining on tumor infiltrating lymphocytes (×400)).

### 2.3 Statistical analysis

Descriptive analyses of the study group were given as numbers and percentages. SPSS Statistics v: 22.0 (IBM SPSS, 2013, Armonk, NY, USA) was used for statistical analysis. Comparisons between recurrent and nonrecurrent cases were made using the Chi-square and Fisher’s Exact test, and the comparison of the Group 1 and Group 2 with the Mc Nemar test. The mean and standard deviation values were compared with student t test. In all analyses, the statistical significance level was taken as p = 0.05. The effects of recurrence, PD-L1 expression, MMR status, and loss of PMS2 on survival in patients were examined using log-rank test. Survival rates were calculated using the Kaplan–Meier survival analysis.

## 3. Results

Of the 74 GBM patients, 29 (39.2%) were female and 45 (60.8%) were male with a median age of 58.4 (range, 4–85). However, the mean age of diagnosis in recurrent cases was 52.1 (4–75) and 62.1 (42–85) in nonrecurrent cases and was significantly lower in recurrent GBM cases (p = 0.007). Twenty-seven cases (36.5%) recurred, 62 cases (83.8%) died, and 12 cases (25.5%) are still alive. The mean recurrence time was 8.2 months (0.8–39.7). Median survival was 9.9 months (6.1–13.8) in all cases from the time of diagnosis, and 12.5 months in recurrent cases; 6 months for nonrecurrent ones. The clinicopathological characteristics of the patients are given in Table 1.

**Table 1:** The clinicopathological characteristics of the recurrent and nonrecurrent cases.

Clinical parameters	Recurrent cases(n = 27)	Nonrecurrent cases(n = 47)	Total(n = 74)
Sex (female/male) n (%)	11 (40.7)/16 (59.3)	18 (38.3)/29 (61.7)	29 (39.2)/45 (60.8)
Mean age (± SD)	52.1 (± 15.349)	62.1 (± 10.693)	58.4 (± 13.392)
Mean recurrence time (month) (95% CI)	8.2 (0.8–39.7)	-	-
Median survival time (month) (95% CI)	12.5 (7.4–17.5)	6.0 (3.7–8.2)	9.9 (6.1–13.8)
Death n (%)	27 (100)	35 (74.5)	62 (83.8)

Student t test: p = 0.02.

**Figure 3:**
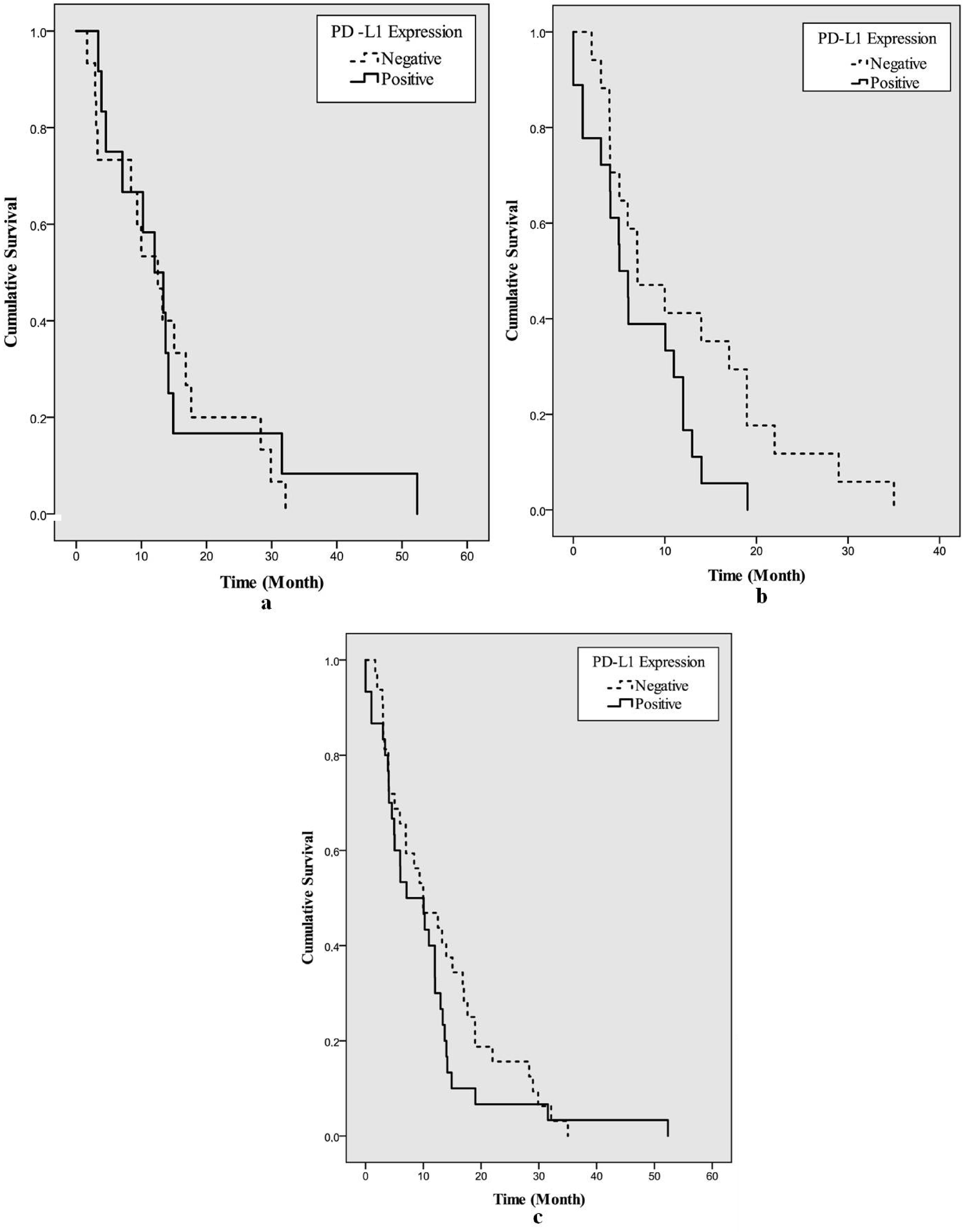


On immunohistochemical study, PD-L1 expression was observed in 36 (48.6%) of the 74 cases. Expression was detected in 12 (44.5%) of the recurrent cases and 24 (51.0%) of the nonrecurrent cases. Median survival was 7.1 months in patients with PD-L1 (+) and 10 months in patients with PD-L1(-). The effect of PD-L1 expression on survival was not significant (p = 0.300), shown in Figure 3 (Figure 3: a) Recurrent cases b) Nonrecurrent cases c) All cases). Thirteen (17.6%) patients exhibited loss of expression for at least one MMR protein and they were considered to MSI. Nine of the cases with MSI were recurrent (33.3%) and 4 were nonrecurrent GBM (8.5%). The details of the immunohistochemical expression of MMR proteins are given in Tables 2 and 3. Median survival for tumors with MSI was 7 months, and 10 months for those with MSS. However, MMR status did not have a significant effect on survival (p = 0.953). Among the MMR proteins, loss of PMS2 was noted in 9 recurrent cases (33.3%) and in 4 nonrecurrent cases (8.5%). Median survival was 7 months in patients with PMS2 loss and 10 months in patients without loss. However, there was no significant effect of PMS2 loss on survival (p = 0.953). Loss of PMS2 was found to be significantly effective in relapsed cases (p = 0.003). The median recurrence time was 10.1 months in those with PMS2 loss, and 39.7 months in those without. PD-1 expression was observed in 6 cases (8.1%). It was observed in 3 recurrent cases (11.1%) and 3 nonrecurrent cases (6.3%). The expressions of PD-L1, PD-1 and presence of MSI are given in Table 4.

**Table 2:** Loss of MMR proteins in the recurrent cases (n = 27, p < 0.05).

MMR proteins	Resection material	n	%	p
MLH1	1st resection	1	3.7	-
	2nd resection	0	0	
MSH2	1st resection	0	0	-
	2nd resection	0	0	
MSH6	1st resection	0	0	-
	2nd resection	2	7.4	
PMS2	1st resection	5	18.5	1.000
	2nd resection	6	22.2	

MMR: Mismatch repair.

**Table 3:** Loss of MMR proteins in all cases (n = 74, p < 0.05).

MMR proteins	Cases	n	%	p
MLH1	Recurrent	1	3.7	0.635
	Nonrecurrent	1	2.1	
MSH2	Recurrent	0	0	-
	Nonrecurrent	0	0	
MSH6	Recurrent	2	7.4	0.530
	Nonrecurrent	2	4.3	
PMS2	Recurrent	9	33.3	0.011*
	Nonrecurrent	4	8.5	

MMR: Mismatch repair.

**Table 4:** The expressions of PD-L1, PD-1, and presence of MSI in the recurrent and non-recurrent cases (n = 74, p < 0.05).

Protein expression	Cases	n	%	p
PD-L1	Recurrent	12	44.5	0.060
	Nonrecurrent	24	51.0	
PD-1	Recurrent	3	11.1	0.536
	Nonrecurrent	3	6.4	
MSI	Recurrent	9	33.3	0.011*
	Nonrecurrent	4	8.5	

MSI: Microsatellite instability.

Clinical features of Group 1 and 2 (recurrent) cases: 11 (40.7%) were female and 16 (59.3%) were male with the median age was 52.1 (range, 4–75). All of them died. The number of cases with PD-L1 (+) in Group 1 was 8 (29.6%). MSI was detected in 6 of 27 cases (22.2%). The number of the cases with PD-1 (+) in Group 1 was 1 (3.7%). Loss of PMS2 was observed in 5 cases (18.5%). The number of cases with PD-L1 (+) in Group 2 was 8 (29.6%), of which 4 were the same cases in Group 1. MSI was detected in 6 of 27 cases (22.2%). Three of these cases were the same as Group 1. The number of cases with PD-1 (+) was 2 (7.4%), both cases were different from Group 1. Six cases had PMS2 loss (22.2%). Two cases were the same as Group 1 and PMS2 loss was observed in 9 cases totally. Clinical characteristics of Group 3 cases: 18 (38.3%) were women and 29 (61.7%) were men with the median age was 62.1 (42–85). Thirty five cases (74.5%) died and 12 cases (25.5%) are still alive.

**Figure 4:**
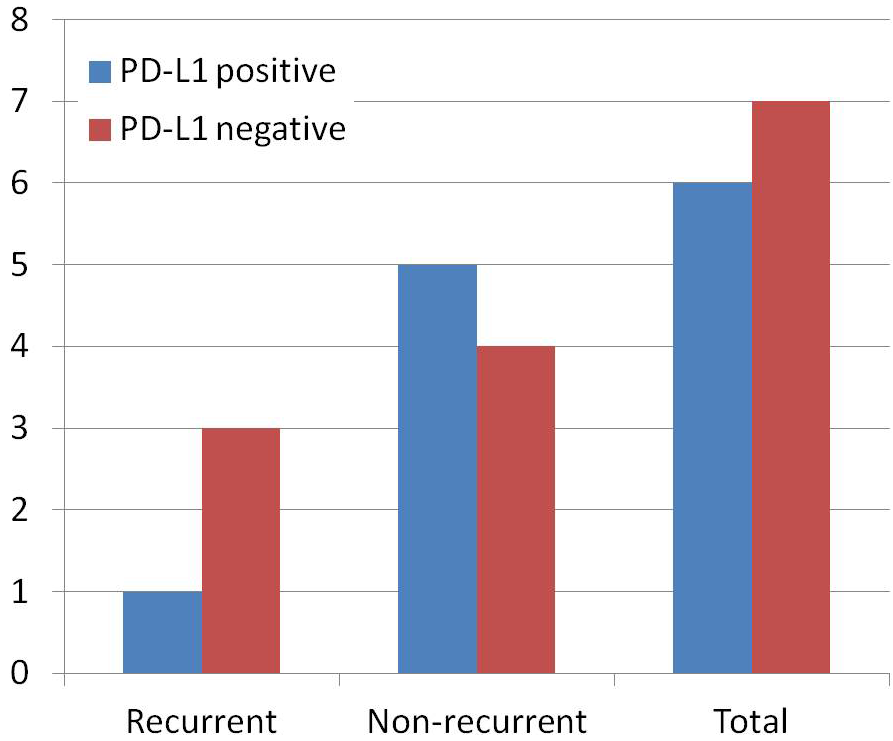


The relationship between MMR status and PD-L1 expression in recurrent and nonrecurrent cases was shown in Figure 4 (p = 0.448 and p = 0.348, respectively, Chi-square Fisher’s exact test). There was no significant difference between PMS2 and PD-L1 expression in patients with or without recurrence (p = 1.000 and p = 0.348, respectively, Chi-square Fisher’s exact test). There was no significant difference between PD-L1 expression and survival in patients with or without recurrence (p = 0.136, log rank (Mantel Cox) test). There was no significant difference between MMR status and survival in patients with or without recurrence (p = 0.133, log rank (Mantel Cox) test). There was no significant difference between loss of PMS2 and survival in patients with or without recurrence (p = 0.133, log rank (Mantel Cox) test).

## 4. Discussion

The ability of GBM to cause local and systemic immunosuppression limits the innate defense and adaptive immunotherapy effect against the tumor, and thus prevents the development of new therapies. The process of immunosuppression is not only related to abnormal PD-L1 expression in GBM cells, but also to the tumor microenvironment.

In one of the most recent studies to elucidate immunotherapy resistance mechanisms in GBM, it was seen that low PD-L1 expression, low tumor mutation burden and T lymphocytes, which are largely depleted in the tumor, are indicators of decreased antitumor immunity [21]. 

However, PD-L1 expression levels are observed in a highly variable range in GBMs. Although it was seen between 61% and 88% in two studies with large patient groups, the median percentage of PD-L1 expression on tumor cells in the study of Nudom et al. was 2.7% [13–15]. Berghof et al. said that the rate of PD-L1-positive cases in GBM was quite higher (72% in recurrent; 88% in newly diagnosed GBM) than melanoma cases (30%) and nonsmall cell lung cancer cases (25%–36%) [13]. In recurrent GBM cases with higher mutations and where immunotherapy resistance is expected less, we also found PD-L1 expression lower than those which are nonrecurrent (44.5% and 51.0%, respectively), similar to the studies of Berghof, Heynckes, and Ndom [13,14,18].

While low PD-L1 expression levels are associated with treatment resistance, some studies have shown that high PD-L1 levels are associated with shorter overall survival in glioma patients [12,17,29]. On the other hand, in several other studies no significant relationship was found between PD-L1 expression and survival [12,13,16,30]. In our study, no significant difference was found between PD-L1 expression level and survival.

In tumors with deficient MMR, 10 to 100 times more somatic mutations were found compared to those which are proficient [21,31]. Microsatellite instability is not high in GBM. Patients with MSI are generally young and have colorectal cancer at the same time [32]. In a study conducted with 30 different tumors, it was stated that the neontigen burden in GBM was in the lower third section [21,33]. In GBM, mutations in MMR genes are thought to be associated with resistance to therapy and thus tumor recurrence [8]. Martine et al. observed that the presence of MSI was at a significantly higher rate in patients with recurrent GBM and stated that this may be associated with malignant progression [34]. We have also found the rate of MSI significantly higher in recurrent patients than the nonrecurrent ones (33.3%, and 8.5% respectively).

GBM specimens containing MSH6 mutations have been described as hypermutator phenotypes [35,36]. Shinsato et al. found reduced levels of MLH1 and PMS2 related to therapy resistance and recurrence [37]. We have also found a significant elevation in PMS2 loss in all groups (Table 2–3). PMS2 loss, which was observed more clearly in recurrent cases, suggested that this change might be a marker for malignant progression. However, we could not find a significant relationship between the loss of PMS2 neither with survival nor PD-L1 expression.

In one of the studies on the role of the status of MSI in predicting immunotherapy response, it was observed that colorectal cancers with MMR deficiency had a high response to PD-1 inhibitor therapy [30]. In another study, the research was expanded and the effectiveness of PD-1 blockade was evaluated in 12 different tumor types with MMR deficiency at an advanced stage and it was seen that 21% of the patients had a complete response and 53% of the patients had an an objective radiological response [38]. In high-grade urothelial carcinomas, it was shown that MMR deficiency (loss of MSH2 and MSH6) is associated with increased PD-L1 expression [8,39]. PD-L1 and PD-L1 expressions were found high in colorectal and endometrial cancers with microsatellite instability (MSI) [8,40]. 

In conclusion, MMR status is suggested as a marker for response to PD-1/PD-L1 blockade in other cancer types. However, we found that PD-L1 expression was low and MSI rate was higher in recurrent GBM than nonrecurrent cases and we could not find a significant relationship between these two entities. The presence of higher MSI in the patients with recurrent GBM in this study indicates the importance of these proteins as a predictive markers. However, low PD-L1 expression levels suggest that this antibody may not be a good predictive marker for determining the group of patients who will receive immunotherapy.

In recent years, an increasing number of clinical trials are available to try different combinations in GBM treatment. We also think that different combinations with other immune checkpoint proteins can be tried in GBM patients to determine both prognostic and therapeutic efficacy.

The limitation of our study was that the clinical data about treatment modalities are incomplete and, therefore, were not included in the article. 
